# Comparison between Bosch and STiGer Processes for Deep Silicon Etching

**DOI:** 10.3390/mi12101143

**Published:** 2021-09-23

**Authors:** Thomas Tillocher, Jack Nos, Gaëlle Antoun, Philippe Lefaucheux, Mohamed Boufnichel, Rémi Dussart

**Affiliations:** 1 Research Group in the Energetics of Ionized Media (GREMI), University of Orléans, CNRS, 14 Rue d’Issoudun BP 6744, 45067 Orléans, France; jack.nos@univ-orleans.fr (J.N.); gaelle.antoun@univ-orleans.fr (G.A.); philippe.lefaucheux@univ-orleans.fr (P.L.); remi.dussart@univ-orleans.fr (R.D.); 2 STMicroelectronics, 10 Rue Thalès de Milet, 37100 Tours, France; mohamed.boufnichel@st.com

**Keywords:** cryogenic etching, STiGer process, Bosch process, plasma etching, time-multiplexed process

## Abstract

The cryogenic process is well known to etch high aspect ratio features in silicon with smooth sidewalls. A time-multiplexed cryogenic process, called STiGer, was developed in 2006 and patented. Like the Bosch process, it consists in repeating cycles composed of an isotropic etching step followed by a passivation step. If the etching step is similar for both processes, the passivation step is a SiF_4_/O_2_ plasma that efficiently deposits a SiO_x_F_y_ layer on the sidewalls only if the substrate is cooled at cryogenic temperature. In this paper, it is shown that the STiGer process can achieve profiles and performances equivalent to the Bosch process. However, since sidewall passivation is achieved with polymer free plasma chemistry, less frequent chamber cleaning is necessary, which contributes to increase the throughput.

## 1. Introduction

Deep silicon etching, also called silicon DRIE (Deep Reactive Ion Etching), has become an essential process step in micromachining, where high aspect ratio structures are often required. The most popular applications are MEMS devices, microfluidics and microelectronics, like TSV (Through Silicon Vias) for the latter. Since features etched with fluorine-based plasmas, which provide the highest average etch rates, have inherently isotropic profiles, passivating the sidewalls is mandatory to achieve anisotropy. The two main DRIE processes, the Bosch process and the cryogenic process, which have been used for about 3 decades, exploit this technique in different ways.

The Bosch process is time-multiplexed, which consists in repeating cycles comprising an etch step and a passivation step [1,2,3,4,5,6,7]. In most cases, the etch step is isotropic and achieved with an SF_6_ plasma. In the passivation step, all the etched surface is coated with a fluoropolymer layer deposited by a C_4_F_8_ plasma (note that other fluorocarbon chemistries are also possible [2,7,8,9]). In each subsequent cycle, ions break though the passivation layer at the etch front and, from one cycle to the other, an isotropic cavity is etched underneath the previous one. This eventually leads to anisotropic profiles with a characteristic roughness at the sidewalls known as “scalloping”. The amplitude of these ripples is usually kept to a minimum value by reducing the etch step time. Such a roughness can be an issue where smooth sidewalls are required. In addition, the polymer layer is also deposited on the chamber walls and, therefore, periodic cleaning procedures are necessary to keep Bosch processes reproducible. Removing the passivation layer from the feature sidewalls may also be critical for several microelectronics applications like TSV [4]. Despite these well-known drawbacks, the Bosch process is famous for its robustness and its stability. For these reasons, it has been used for years in the industry.

The cryogenic process is the result of experiments carried out in the late eighties aiming at freezing chemical reactions on the feature sidewalls by cooling the substrate to a cryogenic temperature [10,11]. Anisotropic profiles were successfully etched in silicon with a SF_6_ plasma, but it was later demonstrated that this result was due to a SiO_x_F_y_ passivation layer formed in the presence of oxygen impurities [12]. Therefore, in cryoetching, a silicon substrate cooled at temperatures around −100 °C is etched with a monocyclic SF_6_/O_2_ plasma. The passivation layer, that is continuously present at the sidewalls and renewed, prevents lateral etching, which leads to high aspect ratio structures with smooth sidewalls. Besides this first advantage, the cryogenic process is faster, since passivation and etching happen at the same time and it is polymer-free. However, in spite of these very interesting points, cryoetching is barely utilized because of the consumption of liquid nitrogen, unwanted in the industry, and reproducibility is sometimes difficult to achieve [5]. In addition, the substrate holder needs to be very uniformly cooled.

These points were highlighted in several papers where the Bosch process and cryoetching were compared [4,5,7,13]. It was also stated that, even if the fluoropolymer layer often needs to be removed from the sidewalls, its higher thickness better helps to limit defects like bowing, as compared to the cryogenic process [4,7]. In addition, the Aspect Ratio Dependent Etching (ARDE) effect is reported to be lower with the Bosch process [4,7]. Selectivity of mask materials has also been discussed. Since the cryogenic process can be operated with lower self-bias voltages than the Bosch process, the selectivity to hard masks (silicon oxide, metal) is usually higher with the cryogenic process. For resist masks, lowering the temperature contributes to increase the selectivity, but the presence of oxygen in the plasma can cause some erosion, which explains a selectivity to resist usually lower than for hard masks [13]. Besides these process-related aspects, Walker et al. also discussed the hardware configuration. Whereas the cryogenic process obviously requires a liquid nitrogen cooling system, the Bosch process needs a fast switching gas injection system as well as fast pumps. Moreover, both chamber walls and turbomolecular pump have to be heated to limit fluoropolymer deposition [13].

These discussions all show that both DRIE techniques have their own advantages. Nevertheless, all the aforementioned papers conclude that the Bosch process is preferred. In spite of more complex etching tools, scalloped sidewalls and polymer contamination, its robustness is better and it does not need liquid nitrogen, which seems to be the most critical points for the users.

In 2006, a new DRIE process, called STiGer, was developed and patented to address the limitations of both the cryogenic and the Bosch processes [14,15]. This is a cryogenic alternated process, relying on a fluoropolymer-free passivation, which presents a better robustness than monocyclic cryoetching. The STiGer process has been compared to the cryogenic process by Tillocher et al. [14], as it will be reminded further, but the Bosch and the STiGer processes have never been compared. This comparison is the main goal of this paper. Firstly, both the principle and features of the STiGer process will be detailed and compared to those of the Bosch process. In parallel, new updates on passivation mechanisms will be incorporated. Then, profiles etched by both the processes of interest will be discussed and compared. Finally, new evolutions of the STiGer process will be presented.

## 2. Materials and Methods

Two ICP (Inductively Coupled Plasma) reactors have been used for the results presented in this article. The first one is an Alcatel 601E ICP reactor already presented in previous publications of the authors [16]. It is composed of a source and a diffusion chamber. The first is a 180 mm in diameter alumina tube with a single-turn antenna wrapped around it. It is driven with a 13.56 MHz power supply with a maximum power of 3 kW. Gases such as SF_6_, SiF_4_ and O_2_, are injected with fast MFC (Mass Flow Controller, minimum gas injection time about 1 s) from the top of the source through a nozzle. Pressure is regulated between 1 and 10 Pa with an APC (Automatic Pressure Control) valve. The substrate holder, located at the bottom of the diffusion chamber, hence about 250 mm from the source, is cooled with liquid nitrogen. Its temperature is regulated with heating resistances between −150 °C and +40 °C. It is powered with a second 13.56 MHz power supply to generate a self-bias voltage.

The second ICP reactor is a PlasmaPro100 Cobra from Oxford Instruments. The source is bigger than that of the previous reactor, with a diameter of 300 mm, and the wafer is slightly closer to the source (230 mm). Both the five-turn antenna and the chuck are powered with 13.56 MHz generators, with maximum powers of respectively 3 kW and 300 W. Gases, in particular SF_6_, SiF_4_ and O_2_, are injected either at the top of the source through a showerhead or through a gas ring located 50 mm above the substrate holder. The MFC of all gases enable fast switching but, moreover, fast ALD valves are installed on the SiF_4_ and O_2_ gas lines, which helps to further decrease the injection time of these gases to a few tens of ms. The temperature of the substrate holder is regulated between −150 °C and 400 °C. Like the first etching tool, liquid nitrogen is used to reach cryogenic temperatures.

For the most of experiments reported in this paper, 6 inches silicon wafers have been used as substrates. For both reactors, they are mechanically clamped on the chuck and a helium backside film is used to enhance the thermal contact. The wafers are (100) oriented and 500 µm thick with two different types of SiO_2_ masks. The first, a 1 µm thick oxide mask, was composed of 0.8 µm trenches. Its loading factor is 20%. The second, also a 1 µm thick oxide mask, was patterned with trenches with critical dimensions from 2 µm to 10 µm and other features that were not used in this study. The loading is low in this last case, about 5%. Only in the case of the profiles displayed in the first figure of part 3.3, the trenches were etched on a 30 mm × 20 mm sample (with the second type of mask) glued on a 6 inches SiO_2_ with a thermal paste.

Etched profiles were observed in cross-section with a Zeiss Supra 40 Scanning Electron Microscope (SEM).

## 3. Results and Discussion

### 3.1. Description of the STiGer Process and First Comparison to the Bosch Process

Like standard cryoetching and the Bosch process, the STiGer process is designed for the deep etching of silicon. It is operated at cryogenic temperature of the substrate, but etch and passivation are alternated. The etch step is usually a short SF_6_ plasma (a few seconds), which leads to shallow isotropic cavities. As explained in the introduction, the passivation step is fluoropolymer-free. It uses a short SiF_4_/O_2_ plasma (also a few seconds) to deposit a SiO_x_F_y_ passivation layer. And, similarly to the cryogenic process, this inhibitor film grows preferentially on cooled surfaces, and most of it desorbs at room temperature. A SiO_x_F_y_ layer can also deposit on the reactor walls at room temperature but its thickness is lower than at cryogenic temperature and it is etched by the periodic SF_6_ plasma [17]. Therefore, contamination is minimal and cleaning is rarely necessary. Since the development of the STiGer process, the understanding of the passivation mechanism has been greatly improved, mostly with two recent publications [17,18]. It is shown by Antoun et al. that SiF_x_ species (with x < 4), produced from the dissociation of SiF_4_ in plasma phase and physisorbing on the substrate surface, have a much longer surface residence time at cryogenic temperature (typically below −65 °C) than at room temperature. Thus, this increases their reaction rate with O radicals, which promotes the growth of the passivation layer. Moreover, XPS characterization has highlighted two additional phenomena [18]. Firstly, the fluorine proportion increases as the temperature decreases such that, at −100 °C, the stoichiometry of the passivation layer is close to SiOF_3_. Secondly, it is in reality a mix of chemisorbed species, a stable fluorinated native oxide, and physisorbed species [18]. When the substrate is heated to room temperature, the physisorbed part of the SiO_x_F_y_ layer is rearranged [18], which eventually leads to the desorption of volatile SiF_4_ as already reported in [19].

**Figure 1:**
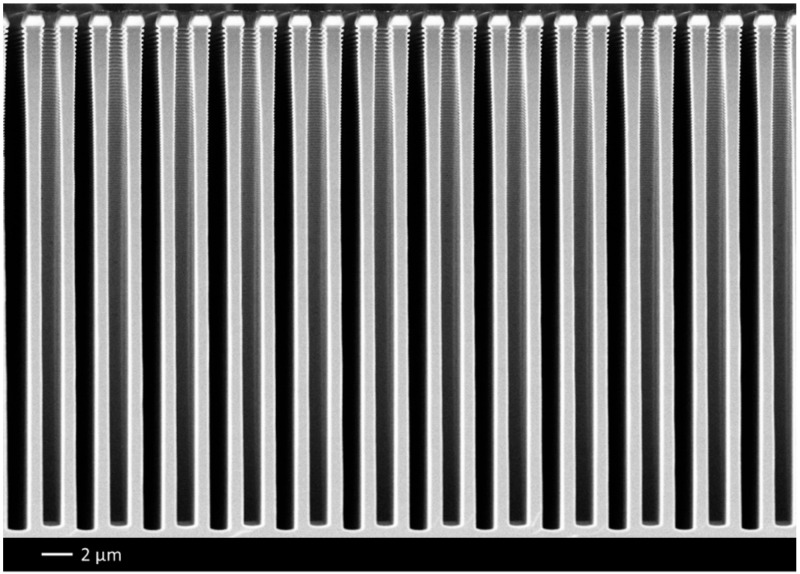
Deep trenches etched with the STiGer process—1 µm SiO_2_ mask, CD = 800 nm, depth = 30.7 µm—1.5 s passivation (50 sccm SiF_4_, 50 sccm O_2_, 1500 W source, 30 V bias, 1.5 Pa), 3.5 s etch (150 sccm SF_6_, 1500 W source, 90 V bias, 3 Pa), 240 cycles, T = −115 °C, Alcatel 601E ICP reactor.

Finally, since a self-bias voltage is applied to the substrate during each etching step, the passivation layer is cleared at the etch front. Then, by repeating etch-passivation cycles as many times as needed, isotropic cavities are etched below the previous ones, leading to anisotropic profiles, like that displayed in Figure 1 as an example. Note that the trenches do not have exactly the same depth because of their staggered arrangement on the mask. Therefore, some of them are cleaved in the middle and others near their end (which can be distinguished on the SEM image in Figure 1). For the latter, this reduces the transport of etchant radicals in the features and, as a result, the trenches are slightly shallower.

Like the Bosch process, the sidewalls are scalloped, which is characteristic of time-multiplexed processes. Compared to the standard cryogenic process, the STiGer process is much more robust to temperature variations. It has been shown in [14] that the profile is almost independent of the temperature over a range of 20 °C, whereas it varies from positive slope to negative slope in the case of standard cryoetching.

Note that an SF_6_/O_2_ plasma can be used in the etch step and, at cryogenic temperature, anisotropic cavities are directly etched [14]. Therefore, there is almost no scalloping and the etch step time can be increased. Such a process, which is an intermediate between the STiGer process and the standard cryogenic process, can also be considered as standard cryoetching process with periodic passivation steps. Therefore, profiles etched with such a process present less scalloping but, according to ref. [14], the temperature stability range is reduced to 10 °C. In the following, only the STiGer process without O_2_ in the etching step will be considered.

The STiGer process parameters have been discussed in [20,21]. In [21], the author first investigated the passivation step with cavity test experiments. She showed that, within the experimental conditions reported in the manuscript, passivation is enhanced by a lower SiF_4_/O_2_ plasma pressure, a higher total gas flow and a higher source power (in the case of ICP reactors). For the Bosch process, the trends are similar for both the C_4_F_8_ gas flow (hence the total gas flow for a single gas) and the source power [3,22,23]. For the Bosch process, passivation also depends on the self-bias voltage since, at larger bias, sputtering tends to decrease the thickness of the passivation layer [3]. The role of pressure is not straightforward. Chen et al. state that the polymer layer is thicker at higher pressure [24]. However, Labelle et al. show that the thickness of the polymer goes through a minimum versus pressure [23]. Thus, on one hand, it increases versus pressure, and it is shown that the layer is more etch resistant with a lower F:C ratio. On the other hand, the authors show that the passivation layer is even thicker as the pressure decreases, which is more similar to the behavior of passivation in the STiGer process. Still regarding the passivation layer in this process, L. Pichon also studied the influence of the SiF_4_/O_2_ gas flow ratio [21]. She showed a more efficient passivation by decreasing this ratio. In this case, this is not only related to the thickness of the passivation layer since it increases by decreasing the SiF_4_/O_2_ ratio [17]. It is rather due to the higher O proportion in the layer which makes it more etch resistant.

The overall behavior of the STiGer process, which is time-multiplexed, is fairly similar to that of the Bosch process. Obviously, profiles are affected in the same way by the etch time to passivation time ratio: it is well known that the achievement of anisotropic features results from a fine balance between passivation and etching steps. Both the etch rate and the sidewall roughness (scalloping) are higher for a longer etch step time at constant passivation time [20,25]. Likewise, rising the self-bias voltage (via the power at the substrate holder), and hence the ion bombardment energy, produces the same effect since it contributes to switch from passivation to an etch regime [25]. Conversely, when passivation dominates, hence at longer passivation time or lower self-bias voltage, the passivation film is not entirely cleared at the etch front. This typically leads to positive profiles with specific defects. This last point is discussed at the end of this part.

The profile slope can be tuned with pressure. In the STiGer process, the position of the APC valve is maintained constant all along the cycles. Therefore, the pressure is varied at the same time in the etching and passivation steps. Decreasing the pressure makes the feature sidewalls change from positive to vertical and even negative slope if it is further decreased [20]. Such a trend was also observed for the Bosch process and attributed to a more directional ion bombardment at decreasing pressure [22,24,26,27]. In addition, at the lowest pressures, passivation becomes less significant than etching and, as feature depth increases, the isotropic cavities enlarge, which eventually leads to negatives profiles.

**Figure 2:**
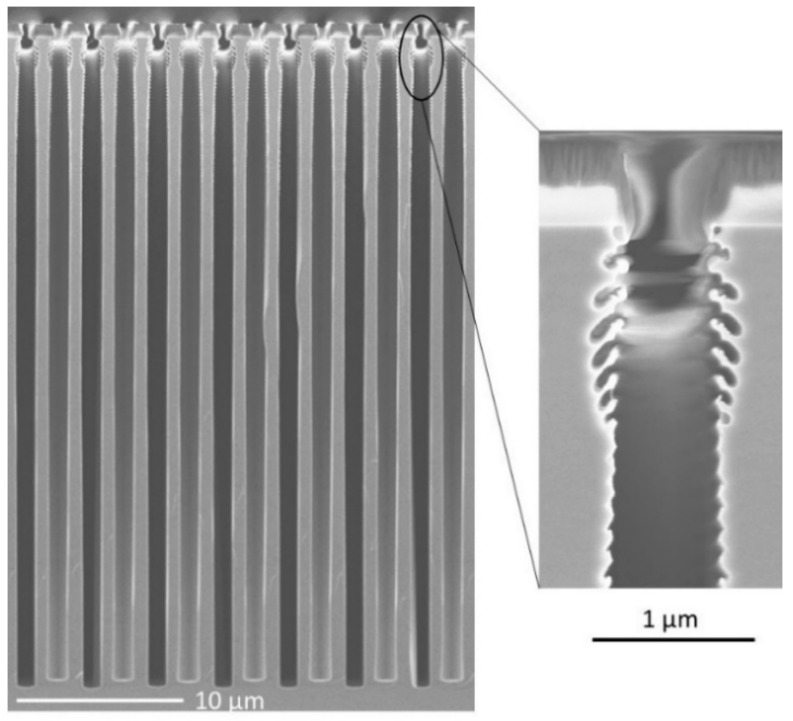
Extended scalloping visible at the top of deep trenches etched with the STiGer process—1 µm SiO_2_ mask, CD = 800 nm, depth = 39.5 µm—1.5 s passivation (50 sccm SiF_4_, 50 sccm O_2_, 1500 W source, 30 V bias, 1.5 Pa), 3.5 s etch (150 sccm SF_6_, 1500 W source, 90 V bias, 3 Pa), 360 cycles, T = −115 °C, Alcatel 601E ICP reactor.

Since it alternates isotropic etch and passivation steps, features etched with the STiGer process exhibit scalloped sidewalls. It was even shown by the author of [25] that an extended scalloping can appear at sufficiently high aspect ratio (>10). This defect corresponds to anisotropic cavities formed on the feature sidewalls, a few micrometers below the mask, as shown in Figure 2.

It was found to originate from ions deviated from their initial trajectory by the edge of the positively tapered mask. Then, these ions can hit the sharp edge of the first (from the top) isotropic cavities and they are subsequently reflected to the opposite sidewall where they remove locally the passivation layer. Finally, anisotropic cavities are etched this way and oriented in direction of the ion bombardment. This extended scalloping is superimposed to the “regular” scalloping along the region where ions are able to hit the sidewalls. This is why the defect is rather located a few micrometers below the mask.

Two methods were proposed to reduce this defect [25]. The first consists in adding a low oxygen flow in the etch cycle, which turns to be the second version of the STiGer process described at the beginning of this section. The second technique consists in gradually increasing the SF_6_ flow in the etching steps, during the first minutes of the process. Consequently, the process starts in a regime where passivation is favored, which helps to limit the extended scalloping.

To our best knowledge, such an extended scalloping has never been reported in the literature for the Bosch process. Nevertheless, some increased sidewall roughness or damage has been reported under specific circumstances by several authors [24,26,28,29,30,31]. It is, in most cases, due to non-normal ion bombardment or when the sidewalls are positive [26,28], which weakens the passivation layer and enhances locally the etch. Choi et al. also reported some sponge-like roughness that was attributed to voids in the deposited polymer or a too thin layer [29]. In all cases, the amplitude of this roughness is not as high as the extended scalloping but, as reminded above, it is possible to prevent extended scalloping with a more robust passivation layer.

### 3.2. Comparison of Profiles Etched by the STiGer and the Bosch Processes

After the previous discussion, based on typical but general trends, the comparison between the STiGer and the Bosch processes can be pushed further by directly comparing feature profiles etched by each of them with equivalent process parameters.

The first ICP reactor presented in Section 2 (Alcatel 601E) was used to etch 4 µm wide trenches with the two processes. The parameters are presented in Table 1.

Both processes were optimized such that anisotropic profiles were etched with parameters as close as possible. Note that the etch rate was not optimized. Therefore, source power, SF_6_ gas flow, etch step time as well as the number of cycles are identical in each case. Passivation parameters were adjusted to balance etching at the sidewalls and hence prevent lateral etching. C_4_F_8_ gas flow for polymerization in the Bosch process and SiF_4_/O_2_ gas flows for passivation in the STiGer process have been set to very close values. Step times are also similar. The self-bias voltages were tuned to 80 V in the etch steps to depassivate efficiently the etch front. In the passivation step of the Bosch process, the bias had to be increased to 105 V to prevent overpassivation. The APC valve, which was fixed all along the processes, was adjusted so that close values of pressure could be reached for both the passivation and the etch steps and for both processes. Of course, the substrate temperature was different in the two cases because one process operates at room temperature and the other at cryogenic temperature.

**Figure 3:**
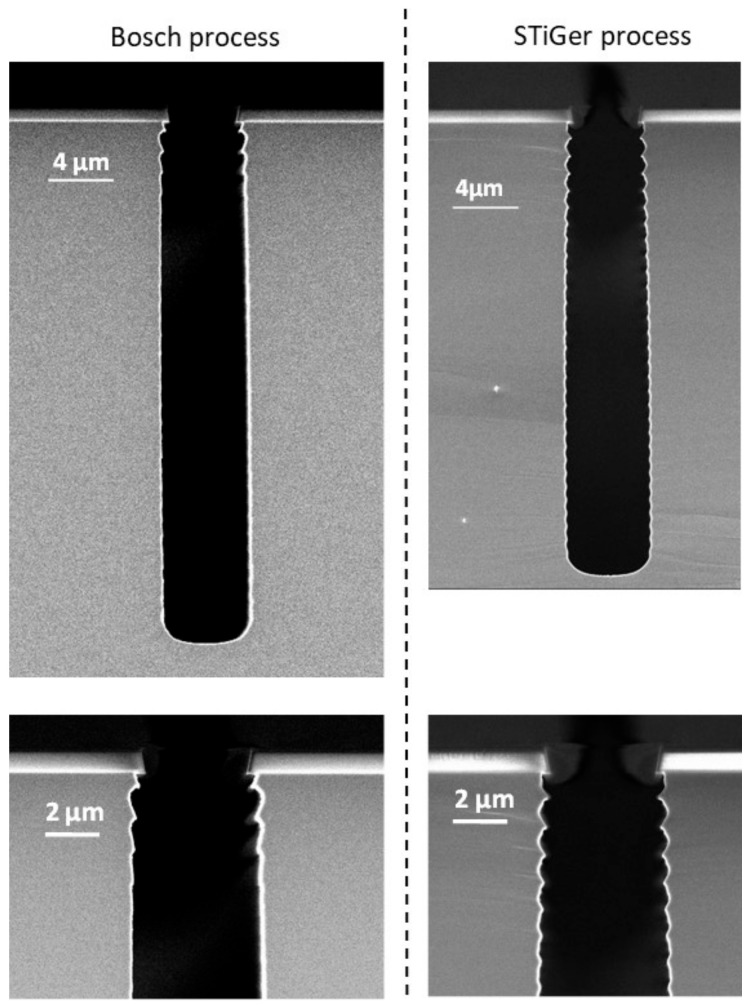
SEM cross-section views of 4 µm trenches etched with the Bosch process (**left**) and the STiGer processes (**right**) using plasmas parameters given in Table 1.

The SEM images of 4 µm trench profiles, etched with the processes described in Table 1, are displayed in Figure 3 and the related measurement are provided in Table 2.

These two profiles are vertical and the one etched with the Bosch process is slightly deeper. However, the etch rate is a little bit higher in the case of cryoetching. Indeed, the difference in depth is compensated by a shorter passivation time for the STiGer process. In addition, it has been shown by Tinck et al. that the silicon etch rate is higher at cryogenic temperature [32].

The selectivity is much larger for the STiGer process, which is due to the lower self-bias voltage in the passivation step. Both profiles exhibit standard scalloping, and it is clear from the SEM images that the scalloping period decreases with the depth, which is a known phenomenon [30,33]. The depth of each isotropic cavity decreases with depth with respect to the ARDE effect (or RIE lag).

However, the sidewalls look slightly smoother in the case of the trench etched with the Bosch process, where the scalloping has almost disappeared 5 µm below the mask and deeper. This difference might be due to the slightly higher etch rate for the STiGer process as well as the shorter passivation step time in the case of the STiGer process. We note that the aspect ratio is not high enough to make extended scalloping appear in the case of the STiGer process.

Therefore, for the specific processes chosen for this comparison, the results are rather comparable. The differences lie in the technique itself. The Bosch process operates at room temperature, thus liquid nitrogen is not necessary, but periodic cleaning steps are needed between each wafer batch because of polymer deposition. Consequently, the STiGer process, which operates at cryogenic temperature but is polymer free, can lead to a slightly higher etch rate as well as a higher throughput.

### 3.3. Perspectives for the STiGer Process

Besides its advantages, several improvements could be beneficial to the STiGer process. As pointed out in the previous parts, minimizing the sidewall roughness, in particular the standard scalloping, is relevant for several applications, like TSV filling. This is directly possible by reducing the cycle time thanks to the progress in the hardware of etching tools (“ultrafast gas-switching”) [9].

**Figure 4:**
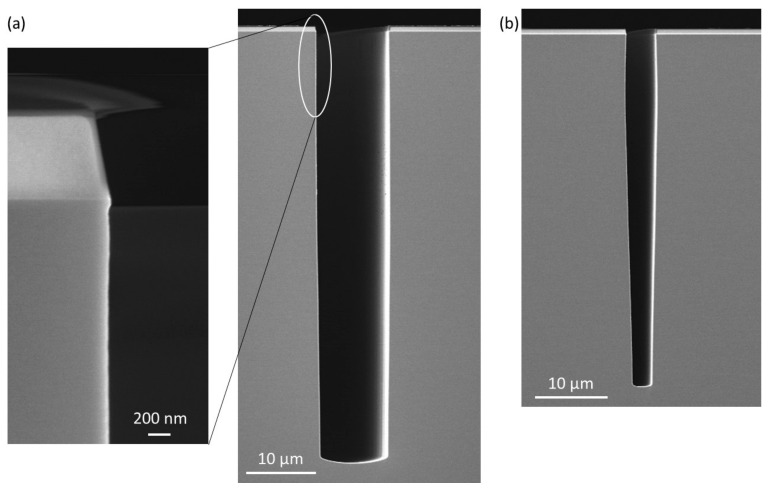
(**a**) Cross section view of 10 µm wide trenches, with magnification of the region below the mask (**b**) Cross section view of 4 µm wide trenches etched with short cycle time STiGer process—50 ms passivation (20 sccm SiF_4_, 20 sccm O_2_, 1500 W source, 15 V bias, 2.2 Pa), 500 ms etch (150 sccm SF_6_, 1500 W source, 95 V bias, 2.5 Pa), 1000 cycles, T = −100 °C, Oxford Instruments ICP reactor.

The Alcatel ICP reactor has been specifically designed to run the Bosch process, and more especially time-multiplexed processes but, as an old generation tool, its performances in terms of cycle time are limited. It is not possible to reach values below 1.5 s. The second reactor described in Section 2, as a state-of-the-art reactor, enables cycle times as low as 10 ms. Therefore, a 1000 cycles process was optimized and run with a passivation time of 50 ms and an etch time of 500 ms. It should be noted that tuning of the process leads to fairly different parameters to those on the previous reactor. The resulting profiles for 10 µm and 4 µm wide trenches are presented in Figure 4 and the parameters are given in the caption.

The 10 µm wide trench has been etched to a depth of 60.8 µm and the 4 µm trench to 49.8 µm, which represents respectively etch rates of 6.6 µm·min^−1^ and 5.4 µm·min^−1^. The slope is slightly positively tapered for the both trenches (89.7° and 89.4° respectively). No significant defects are visible; the undercut at the top of the 10 µm trench (Figure 4a) is negligible. A closer view on the sidewalls (see the magnified view of the top of the trench in Figure 4a), reveals that scalloping has been greatly reduced to a residual roughness. Consequently, and as expected, the cycle time is a relevant knob to minimize the sidewall defects in the STiGer, similarly to what is reported for the Bosch process [9].

The ability to operate the STiGer process without liquid nitrogen is another improvement of interest. Therefore, it is necessary to shift the temperature window to higher values, like −50 °C, which can be reached with a chiller.

However, increasing the temperature that much (by nearly 60 °C), has a significant impact on passivation. As demonstrated by Antoun et al. in their experimental conditions, the composition of the passivation layer is different below and above −65 °C [18]. Below −65 °C, as already reminded previously, the passivation layer is a mix of chemisorbed species and physisorbed species, with a high content of fluorine. Above −65 °C, but below −40 °C, the passivation layer is thinner [17] and only composed of the chemisorbed species, with a fluorine proportion equivalent to that of oxygen [18]. At such higher temperatures, passivation is less efficient and lateral etching appears.

**Figure 5:**
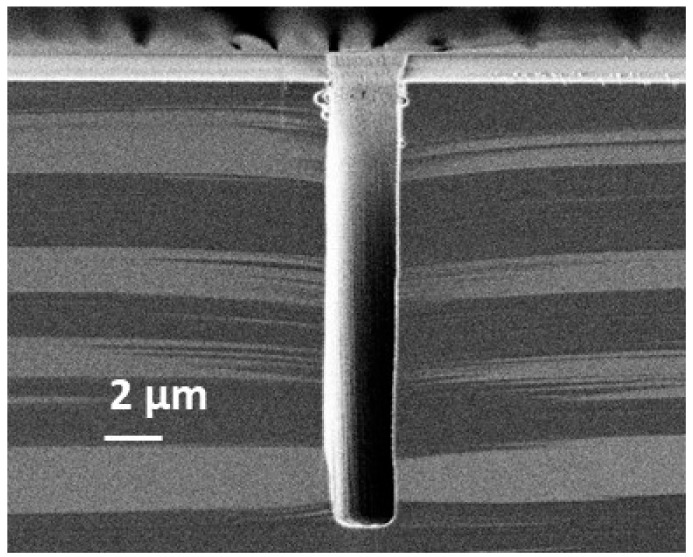
Cross section view of 2µm wide trenches etched at −50 °C with STiGer process—2 s pas-sivation (70 sccm SiF_4_, 70 sccm O_2_, 1500 W source, 15 V bias, 1.1 Pa), 1.5 s etch (150 sccm SF_6_, 1500 W source, 90 V bias, 1.9 Pa), ~171 cycles, T = −50 °C, Alcatel 601E ICP reactor.

Therefore, if the operating temperature range of a cryogenic process is extended to intermediate values like −50 °C, passivation needs to be reinforced. For the STiGer process, this mostly consisted in reducing the etch step time (1.5 s) and increasing the passivation step time (2 s). Thus, compared to the STiGer processes at cryogenic temperature, the passivation time is now higher than that of the etch. An example of a 2 µm wide trench etched under such conditions in the Alcatel 601E reactor (still cooled with liquid nitrogen) is given in Figure 5, as well as its experimental conditions.

The trench profile is anisotropic with some defects, having a maximum amplitude of 600 nm, just below the mask. They suggest a weakness in the passivation layer at this location, which shows that passivation can still be optimized. The etched depth is 16.44 µm, which means an average etch rate of 1.6 µm·min^−1^. The etch rate is lower at this temperature because, now, most of the process time is dedicated to passivation. Selectivity to the oxide mask has been estimated to 160:1, which is higher than for the other examples presented previously in this article. This is due to a lower self-bias voltage and hence a lower ion bombardment.

This process demonstrates the feasibility to operate the STiGer at a temperature where liquid nitrogen is no longer mandatory. This solution can address one of the limitations of the STiGer process compared to the Bosch process.

## 4. Conclusions

Both the Bosch and the STiGer processes are time-multiplexed processes, fairly similar in their base principle. For this reason, etch rates are of the same order of magnitude in comparable experimental conditions. In addition, etch profiles are scalloped in both cases and they show a comparable dependency to the process parameters they have in common. It has been shown that the sidewall roughness can be reduced by decreasing the cycle time.

However, both processes rely on different passivation steps. For the Bosch process, it consists in depositing a fluoropolymer on the sidewalls at room temperature of the substrate. This layer is also deposited everywhere in the reactor, which explains why frequent cleaning procedures are necessary. For the second, the passivation layer is deposited at cryogenic temperature by a SiF_4_/O_2_ plasma. Since it deposits only on cold surfaces, the reactor is much less contaminated.

The Bosch process is well known and widely used in the industry for its robustness. The STiGer process is more recent but presents interesting advantages, like a good stability versus temperature and can lead to a higher throughput since chamber cleaning are rarely required. It usually operates at cryogenic temperature, but it has been shown the perspective to raise the temperature to −50 °C, where liquid nitrogen is no longer necessary. All these points are summarized in Table 3.

## Figures and Tables

**Table 1:** Experimental conditions for both Bosch and STiGer processes, Alcatel 601E ICP reactor.

Parameters	Bosch		STiGer	
	Etching	Passivation	Etching	Passivation
Gas	SF_6_	C_4_F_8_	SF_6_	SiF_4_/O_2_
Flow (sccm)	50	27	50	25/12
Cycle time (s)	15	10	15	7 s
Pressure (Pa)	3.0	2.0	3.9	1.2
Source power (W)	1500	1500	1500	1500
Self-bias voltage (V)	80	105	80	30
Temperature (°C)	21.4 °C		−90.0 °C	
Total time	12 min 30 s		11 min	
Number of cycles	30		30	

**Table 2:** Measurements on trench profiles displayed in Figure 3 and etched with the STiGer and the Bosch processes, Alcatel 601E ICP reactor.

Process	Profile	Depth	Etch Rate (µm/min)	Si:SiO_2_ Selectivity
Bosch process	Vertical	31.2 µm	2.50	138
STiGer process	Vertical	27.8 µm	2.53	185

**Table 3:** Summary of the comparison between Bosch and STiGer processes.

Parameter	Bosch	STiGer
Substrate temperature	Room temperature	T < −50 °C
Passivation chemistry	Fluoropolymer	Fluoropolymer free
Average etch rate	Similar	
Sidewall roughness	Similar (scalloping)	
Process stability versus temperature	Very stable	Stable over a temperature range of 20 °C
Chamber clean	Yes	No
Specific hardware feature	- Heated chamber walls and pumps - Fast MFC	- Liquid nitrogen for T around −100 °C - Low temperature chiller around −50 °C - Fast MFC

## Data Availability

Not applicable.
